# Role of 3D Printing in Preoperative Planning for Spine Surgery: A Scoping Review

**DOI:** 10.7759/cureus.111087

**Published:** 2026-06-18

**Authors:** Abigail Jenkins, Faraaz Azam, Pooja Venkatesh, Ammar Haider, Sanjay V Neerukonda, Sapan Patel, Sruthi Ranganathan, Shubhang Bhalla, Mazin Al Tamimi, Christie Caldwell, Kristen Hall, Umaru Barrie, Salah G Aoun

**Affiliations:** 1 Neurological Surgery, University of Texas Southwestern Medical School, Dallas, USA; 2 Neurological Surgery, University of Texas Southwestern Medical Center, Dallas, USA; 3 Neurological Surgery, Emory University, Atlanta, USA; 4 Internal Medicine, Combined Military Hospital (CMH) Lahore Medical College and Institute of Dentistry, Lahore, PAK; 5 Neurological Surgery, McGovern Medical School, Houston, USA; 6 Neurological Surgery, Rutgers University New Jersey Medical School, Newark, USA; 7 Neurological Surgery, University of Cambridge, Cambridge, GBR; 8 Neurological Surgery, Paul L. Foster School of Medicine, El Paso, USA; 9 Neurological Surgery, New York University (NYU) Langone Health, New York, USA

**Keywords:** 3d, 3d printing, scoping review, spine surgery, surgical planning, technology

## Abstract

Three-dimensional (3D) printing has emerged as a useful adjunct in spine surgery, particularly for preoperative planning, surgical rehearsal, education, patient communication, navigation template development, and selected patient-specific applications. This scoping review maps the available literature on the use of 3D printing in preoperative planning and related applications for spine surgery. The review was conducted according to the Preferred Reporting Items for Systematic Reviews and Meta-Analyses extension for Scoping Reviews (PRISMA-ScR) guidelines using PubMed, Google Scholar, Embase, SCOPUS, and Web of Science. Databases were searched from inception through September 2025. Seventy-one unique articles were included: 12 comparative studies; 20 non-comparative clinical, technical, simulation, or education-focused studies; and 39 additional case reports or technical notes. The retrospective studies were further stratified into 20 non-comparative retrospective studies and 12 comparative retrospective studies evaluating 3D printing-assisted approaches against control or comparator approaches. Across the included literature, 3D printing was most commonly used for anatomical visualization, surgical rehearsal, screw trajectory planning, navigation template or drill guide development, patient education, trainee education, intraoperative reference, and selected custom implant or prosthetic applications. Several comparative studies reported favorable study-level findings associated with 3D printing, including reported improvements in screw placement accuracy or acceptable placement, operative duration, blood loss, fluoroscopy exposure, and complications; however, these outcomes were reported inconsistently and were not pooled quantitatively. Reported limitations included upfront production costs, prolonged preparation time, imaging and segmentation requirements, limited soft-tissue or biomechanical realism, and the need for specialized software, equipment, and personnel. Overall, 3D printing appears to be a promising adjunct for selected spine surgery applications, but current evidence remains largely retrospective, heterogeneous, and descriptive. Prospective, multicenter, controlled studies with standardized outcome measures, cost-effectiveness analyses, and longer follow-up are needed before 3D printing can be recommended as a standard component of preoperative planning in spine surgery.

## Introduction and background

Standard preoperative planning in spine surgery typically relies on radiographs, computed tomography (CT), magnetic resonance imaging (MRI), multiplanar image review, virtual three-dimensional (3D) reconstructions, and, in selected cases, intraoperative navigation or robotic guidance. These tools remain essential and form the foundation of modern spine surgery planning. However, surgeons must often mentally translate two-dimensional or screen-based images into 3D operative anatomy, which can be challenging in cases involving severe deformity, craniovertebral junction anomalies, congenital abnormalities, revision anatomy, trauma, or spinal tumors. These scenarios often involve distorted landmarks, narrow osseous corridors, abnormal screw trajectories, and close proximity to the spinal cord, nerve roots, vertebral arteries, segmental vessels, and other critical structures.

3D printing has evolved from a manufacturing technology into a biomedical and surgical tool with applications across medicine, surgery, and neurosurgery [1-5]. In spine surgery, 3D printing has emerged as a useful adjunct for preoperative planning, surgical rehearsal, patient and trainee education, navigation templates, and patient-specific applications [6-17]. By converting patient imaging into physical models, 3D printing can provide tactile and spatial understanding of complex anatomy, assist with screw trajectory planning, facilitate rehearsal of complex osteotomies or resections, and improve communication among surgeons, trainees, and patients [12,17-24]. In selected complex cases, this may help supplement conventional CT and MRI review by allowing surgeons to directly inspect, manipulate, and simulate procedures on patient-specific anatomy before entering the operating room [24-30].

In this scoping review, 3D printing refers to several related applications, including patient-specific anatomical models for preoperative planning, surgical rehearsal, and intraoperative reference; drill guides and navigation templates for screw placement or osteotomy planning; instrumentation planning tools for screw trajectory selection, implant sizing, and rod contouring; custom implants or prosthetic reconstructions; and simulation models used for trainee or patient education [31-46]. However, barriers including cost, availability, preparation time, and the need for specialized software, equipment, and personnel continue to limit widespread adoption [5,24,32]. Therefore, this review maps the available literature on 3D printing in spine surgery, with emphasis on its applications in preoperative planning, intraoperative guidance, implant customization, and education, while summarizing reported advantages, limitations, and areas requiring further study.

## Review

Materials and methods

Search Strategy

**Figure 1:**
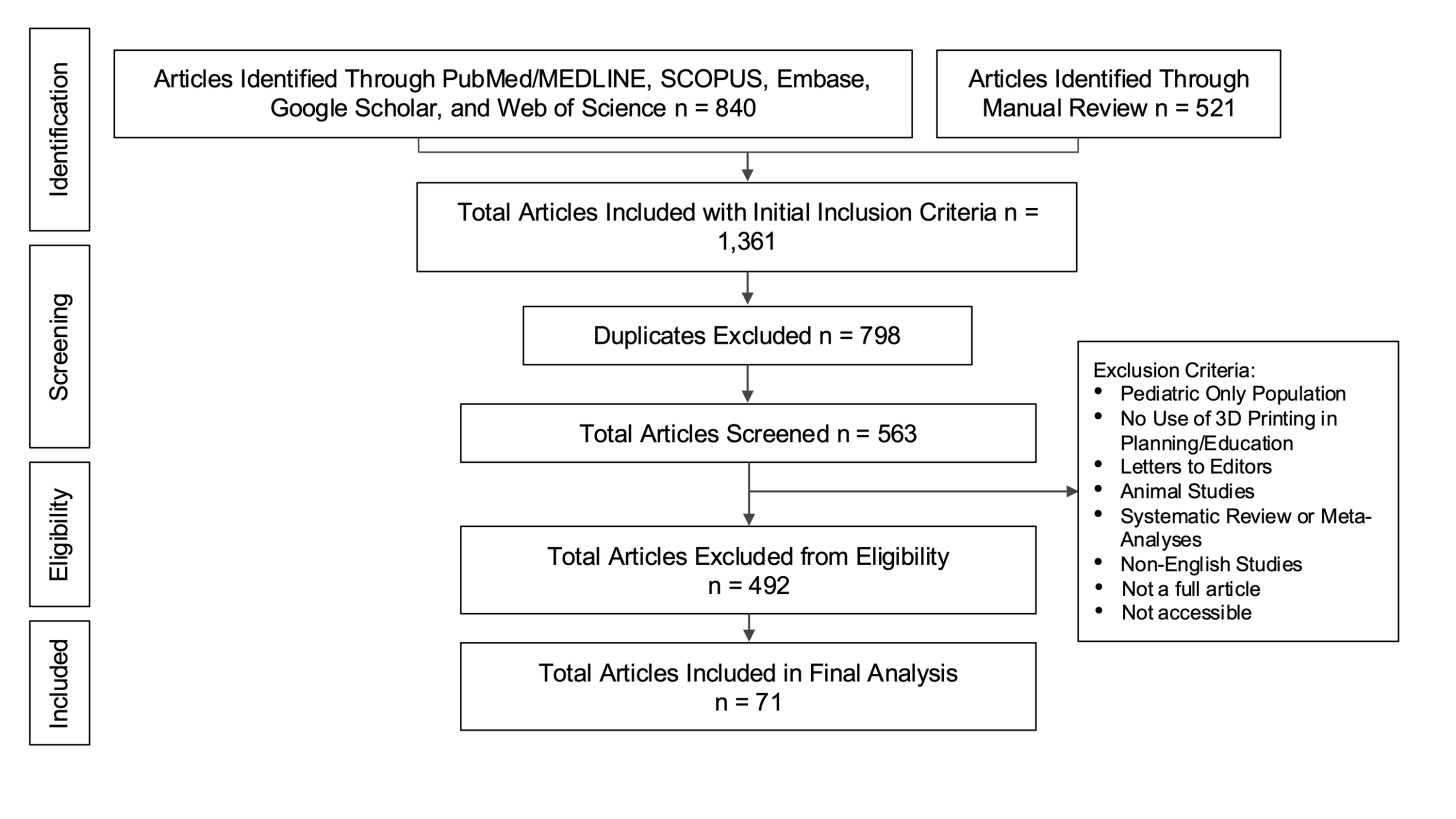
Preferred Reporting Items for Systematic reviews and Meta-Analyses extension for Scoping Reviews (PRISMA-ScR) flow diagram showcasing the methodology employed in conducting the scoping review. 3D: three-dimensional

This scoping review was conducted following the Preferred Reporting Items for Systematic Reviews and Meta-Analyses extension for Scoping Reviews (PRISMA-ScR) guidelines, utilizing PubMed, Google Scholar, Embase, SCOPUS, and Web of Science databases (Figure 1). Database-specific search strategies combined terms related to 3D printing, including “3D printing,” “three-dimensional printing,” “3D printed,” “additive manufacturing,” “rapid prototyping,” “stereolithography,” “patient-specific model,” “printed model,” “navigation template,” and “patient-specific guide,” with spine-related terms including “spine,” “spinal,” “vertebra,” “scoliosis,” “kyphosis,” and “pedicle screw.” No date restrictions were applied, and databases were searched from inception through September 2025. The search was last updated in September 2025, and studies published through September 2025 were included in the current analysis. The full database-specific search strings are provided in the appendix. For Google Scholar, the first 200 results sorted by relevance were screened for each search query.

The inclusion criteria encompassed studies involving adult or mixed-age spine-related cohorts, as well as spine-related procedures or protocols utilizing 3D technology for planning and/or education. Although this review was primarily focused on adult spine surgery, mixed-age or adolescent deformity studies were retained when the 3D printing application involved spine-related preoperative planning, navigation template development, procedural simulation, screw trajectory planning, or education that was directly relevant to adult spine surgery workflows. Because education and simulation are major applications of 3D printing in surgical preparation, studies focused on spine-related anatomical education, procedural simulation, or trainee preparation were included when they involved 3D models relevant to spine surgery or neurosurgical spine anatomy. However, these studies were analyzed separately from clinical preoperative planning studies and were not used to infer clinical perioperative outcomes.

Exclusion criteria included review articles, letters to the editor, meta-analyses, procedures or protocols unrelated to spine surgery, studies lacking 3D technology or preoperative planning/education usage, articles not available in English, and basic science publications without direct relevance to spine-related clinical planning, surgical preparation, anatomical education, procedural simulation, or patient-specific spine modeling. Cadaveric, biomechanical, or finite-element studies were included only when they involved patient-specific or spine-relevant 3D printing applications for planning, simulation, education, or procedural preparation; purely bench-based, non-spine, or non-translational studies were excluded.

Studies limited exclusively to pediatric populations were excluded unless the study involved a mixed-age cohort or a spine-related 3D printing workflow directly relevant to adult-applicable preoperative planning, simulation, education, or patient-specific guide development. Exclusively pediatric studies were excluded because this review was designed to focus primarily on adult spine surgery. Pediatric spine conditions, including congenital scoliosis, spinal dysraphism, and early-onset kyphoscoliosis, often involve distinct growth-related anatomy, surgical indications, implant considerations, perioperative goals, and long-term outcomes compared with adult spine pathology. Excluding pediatric-only studies was therefore intended to reduce clinical heterogeneity and allow for a more focused synthesis of 3D printing applications in adult spine surgery.

A scoping review design was selected rather than a traditional systematic review or meta-analysis because the available literature on 3D printing in spine surgery is heterogeneous with respect to study design, clinical indication, printing technology, application type, and reported outcomes. The goal of this review was therefore to map the breadth of available evidence, categorize the applications of 3D printing in spine surgery, summarize reported advantages and limitations, and identify areas requiring further study rather than to generate a pooled estimate of treatment effect. A protocol for this scoping review was not prospectively registered. However, the review was conducted according to PRISMA-ScR guidance, and the search strategy, eligibility criteria, data extraction variables, and synthesis approach were defined before final data abstraction.

Data Extraction

Two independent authors (A.J. and F.A.) conducted the initial literature search and independently screened titles and abstracts for relevance. Articles meeting inclusion criteria or requiring further review were advanced to full-text assessment. A comprehensive full-text review was then undertaken by eight authors (A.J., F.A., P.V., S.V.N., A.H., S.B., S.P., and S.R.) to confirm eligibility and extract relevant data. The bibliographies of included articles were also reviewed to identify additional studies meeting inclusion criteria. Disagreements regarding article eligibility or extracted data were resolved through discussion and consensus among the reviewing authors, with involvement of the senior author when needed. Bibliographic records, screening decisions, and extracted study data were managed using a standardized electronic spreadsheet.

The following variables were extracted from each included study when available: author, year of publication, country, study design, study aim, sample size, patient demographics, presenting symptoms, clinical diagnosis or pathology, spinal location, imaging modality, type of 3D printing application, type of intervention, surgical or conservative management, operative duration, estimated blood loss, accuracy rate, acceptable rate, angle of deviation, complications, follow-up duration, neurological or clinical outcomes, reported advantages of 3D printing, reported disadvantages or limitations, recommendations, and future directions. For the purposes of data extraction, accuracy rate was defined as the proportion of screws in the optimal location on postoperative imaging as predicted by the simulated location, while acceptable rate referred to screws within safe margins of the optimal location, generally defined as no more than 2.0 mm of variance from the intended position when this threshold was provided. Angle of deviation was defined as the angle of variance between the true screw position as visualized on postoperative imaging and the optimal position simulated preoperatively. However, the included studies varied in their definitions, imaging methods, grading systems, reporting units, and thresholds for these outcomes. Therefore, these variables were abstracted descriptively and were not treated as directly comparable pooled outcomes.

In accordance with PRISMA-ScR guidelines, a formal quality appraisal or risk-of-bias assessment of the included studies was not performed. Because of heterogeneity in study designs, indications, interventions, and reported outcomes, the findings were summarized descriptively using counts, percentages, and narrative synthesis rather than weighted analysis or meta-analysis.

Results

Electronic Search Yield

The primary search, as outlined in Figure 1, yielded 1,361 studies. Following the removal of 798 duplicate studies, 563 articles were screened. After reassessment of eligibility, 492 articles were excluded in accordance with the inclusion and exclusion criteria. The final analysis incorporated 71 unique articles. These included 12 comparative studies [6-17] evaluating 3D printing-assisted approaches against control or comparator approaches; 20 non-comparative clinical, technical, simulation, or education-focused studies [18-37]; and 39 additional case reports, technical notes, case series, protocol studies, or application-focused reports [38-76].

The included studies were organized into three mutually exclusive categories: comparative studies, non-comparative clinical/technical/education-focused studies, and additional case reports, technical notes, case series, protocol studies, or application-focused reports. Across these categories, 3D printing was most commonly used for patient-specific anatomical visualization, screw trajectory planning, surgical rehearsal, navigation template or drill guide development, patient communication, and trainee education. Comparative studies generally evaluated perioperative or technical outcomes such as screw placement accuracy, operative duration, blood loss, fluoroscopy exposure, complications, and patient-reported outcomes. Non-comparative studies more commonly described feasibility, workflow integration, simulation value, patient-specific planning, or educational utility. The additional reports primarily demonstrated application of 3D printing in complex deformity, tumor, trauma, craniovertebral junction pathology, patient-specific implant or guide development, education, simulation, or procedural planning.

Utilization of 3D Printing in the Non-comparative Retrospective Studies

Table 1 outlines the utilization of 3D printing in 20 non-comparative retrospective studies [18-37]. These studies used 3D printing for patient-specific anatomical visualization, preoperative planning, surgical simulation, education/training, screw or trajectory planning, navigation template development, and intraoperative reference. Models were reconstructed from existing imaging modalities, including X-ray in eight studies [20,22,23,27,30,31,34,36], MRI in six studies [22-24,34,35,37], and CT in 16 studies [18,20,22-28,30-34,36,37]. Across the non-comparative retrospective studies, reported applications included preoperative anatomical visualization, surgical rehearsal, screw or trajectory planning, navigation template development, education/training, and patient communication. Education-focused studies, including Ramirez et al. [29], Burkhard et al. [19], Clifton et al. [21], Patchana et al. [28], and Braun et al. [18], were analyzed separately from clinical preoperative planning studies and were not used to infer perioperative efficacy. Clinical studies more commonly described feasibility, anatomical visualization, trajectory planning, intraoperative reference, or patient-specific guide development. Reported limitations included preparation time, cost/resource burden, imaging or radiation requirements, technical or software constraints, limited soft-tissue or biomechanical realism, and limited generalizability. Because study designs, procedures, pathologies, and outcome definitions varied substantially, these findings were summarized descriptively rather than pooled quantitatively.

**Table 1:** 3D printing applications, categorical advantages and limitations, and conclusions in non-comparative retrospective studies that assessed 3D printing for spine surgery planning, simulation, education, or procedural preparation. 3D: three-dimensional; AIS: adolescent idiopathic scoliosis; ATPS: anterior transpedicular screw fixation; CT: computed tomography; CVJ: craniovertebral junction; LMS: lateral mass screw; PS: pedicle screw; MIS-TLIF: minimally invasive transforaminal lumbar interbody fusion; PSG: patient-specific guide; PSO: pedicle subtraction osteotomy; TOLF: thoracic ossification of the ligamentum flavum

#	Author, year, country	Study aim	Use of 3D	Categorical advantages and limitations	Conclusion, recommendations
1	Xin et al., 2022 [35], China	To develop a 3D-printed PSO guide plate system for patients with kyphosis.	Used in the construction and utilization of pedicle, lamina osteotomy, and vertebral osteotomy guide plates.	Advantages: preoperative planning; surgical simulation; osteotomy guidance; trajectory planning. Limitations: limited bone/soft-tissue realism; potential technical deformation.	The new 3D-printed PSO guide plate system demonstrated effective preoperative osteotomy planning with commendable accuracy, laying a foundation for clinical use. Clinical application, osteotomy process, and instrument feasibility should be demonstrated in cadavers or animals for validation.
2	Ramirez et al., 2022 [29], Russia	To introduce a global, affordable 3D neuroanatomy teaching model for hands-on education.	Low-cost 3D acrylonitrile-butadiene styrene pen models and 3D-printed or cadaveric bone frames were used to create hands-on educational models relevant to neuroanatomy and spine-related surgical education.	Advantages: education/training; cost efficiency; surgical simulation; customizable models. Limitations: user-dependent model creation; potential human error.	The technique provides a cost-effective way to create replicable 3D models and may help reduce neuroanatomy and spine surgery education gaps between low- and high-resource facilities.
3	Burkhard et al., 2019 [19], Switzerland	To assess 3D-printed vertebrae for realistic haptic simulation of PS placement and decompression surgery.	Employed 3D-printed vertebral models for simulation of PS placement and decompression.	Advantages: education/training; haptic realism; surgical simulation. Limitations: cost/resource burden; pre- and post-processing requirements.	The parameterizable vertebra model provided realistic haptic simulation for PS insertion and decompression, outperforming Sawbones models and marking progress toward patient-specific 3D-printed spine replicas. Additional biomechanical testing and clinical usability studies are recommended.
4	Stefan et al., 2020 [31], Germany	To introduce a cost-effective 3D printing method for spine surgery simulation using CT-based synthetic bone models.	CT-based synthetic bone models were 3D-printed to replicate cortical and cancellous structures for haptic spine surgery simulation.	Advantages: education/training; realistic anatomy; cost efficiency; fluoroscopy-compatible simulation. Limitations: limited biomechanical validation; sampling limitations.	The low-cost, CT-based 3D-printed spine model accurately replicated haptic properties for surgical simulation and was compatible with fluoroscopy and X-ray imaging. Further mechanical testing, surface optimization, and comparison with human bone are recommended.
5	Govsa et al., 2018 [23], Turkey	To determine entry points for C1 LMS using 3D patient-specific spine models.	Patient-specific 3D C1 fracture models were created to assist in the identification of fracture location, pedicle size, vertebral artery anatomy, and screw entry points.	Advantages: anatomical visualization; preoperative planning; screw trajectory planning; patient-specific modeling. Limitations: none specifically reported.	C1 screw fixation is technically challenging and requires detailed knowledge of cervical anatomy. Patient-specific 3D models may aid anatomical understanding, anticipate technical challenges, and guide optimal surgical approaches.
6	Thayaparan et al., 2020 [33], Australia	To share the experience of using 3D-printed BioModels in 129 consecutive MIS-TLIF cases over 27 months, performed by one surgeon at a single center.	Life-size lumbar spine BioModels created using stereolithography and acrylic resin aided preoperative consultation, surgical planning, implant selection, and patient discussion.	Advantages: preoperative planning; patient education; implant selection; surgical workflow optimization; cost efficiency. Limitations: preparation time; limited use in urgent cases; lack of comparative data.	Patient-specific MIS-TLIF planning with BioModels and the SpineBox process was feasible and may save costs by tailoring implants, equipment, and workflow. Standardized metrics are needed to compare surgical techniques and technology-assisted workflows.
7	Zhao et al., 2017 [37], China	To evaluate clinical outcomes in microsurgical decompression of TOLF via a paraspinal approach using a percutaneous tubular retractor system.	Patient-specific vertebral models were created for precise surgical planning, patient education, and measurement of vertebral fenestration size and location.	Advantages: preoperative planning; anatomical visualization; patient education; surgical guidance. Limitations: none specifically reported.	Patient-specific 3D-printed vertebral models may support microsurgical decompression of TOLF by improving preoperative visualization, facilitating surgical planning, and helping determine the optimal decompression window. Further comparative studies are needed to clarify the added value of 3D-assisted planning.
8	Li et al., 2018 [25], China	To create personalized navigation templates for insertion of lower cervical ATPS using 3D printing.	Individualized lower cervical 3D-printed navigation templates were developed for planning anterior transpedicular screw trajectories.	Advantages: screw trajectory guidance; patient-specific planning; cost efficiency. Limitations: need for template optimization; early-stage clinical application.	Personalized 3D-printed navigation templates showed feasibility for lower cervical anterior transpedicular screw placement. Further optimization of template parameters, anchor points, and coupling structures is needed before broader clinical use.
9	Senkoylu et al., 2020 [30], Turkey	To evaluate the safety and effectiveness of patient-specific 3D rapid-prototype printing for PS insertion in AIS.	Patient-specific 3D-printed PS guides were created for vertebral levels in AIS.	Advantages: screw trajectory guidance; surgical accuracy; safety; reduced intraoperative preparation. Limitations: imaging/radiation requirement; limited comparative evidence.	Patient-specific 3D-printed PS guides may improve the safety and accuracy of PS placement in AIS. Larger comparative studies are needed to validate accuracy, efficiency, radiation-related tradeoffs, and generalizability.
10	Gao et al., 2017 [22], China	To evaluate the feasibility and efficacy of individualized 3D-printed model-assisted posterior internal fixation in treating CVJ abnormalities.	Personalized 3D-printed models aided preoperative evaluation of CVJ abnormalities, surgical simulation, and treatment planning.	Advantages: preoperative planning; surgical simulation; anatomical visualization; safety. Limitations: preparation time; template exposure requirements; limited emergency use.	Individualized 3D-printed models may assist preoperative evaluation, surgical simulation, and posterior internal fixation planning for CVJ abnormalities. Preparation time and exposure requirements may limit use in urgent settings.
11	Sugawara et al., 2017 [32], Japan	To evaluate methods for insertion of C1 LMS, C2 PS, and C2 laminar screws.	Preoperative CT images were used to plan safe screw trajectories and create patient-specific screw guide templates for posterior C1-C2 fixation.	Advantages: screw trajectory guidance; surgical accuracy; cost efficiency; patient-specific planning. Limitations: preparation time; soft-tissue/template fit limitations.	Patient-specific screw guide templates provided an accurate and simplified method for C1-C2 screw insertion and may reduce reliance on complex intraoperative navigation. Further clinical validation and technical refinement are warranted.
12	Leary et al., 2021 [24], USA	To utilize 3D-printed models for preoperative planning and intraoperative guidance in complex spine tumors.	3D-printed models were used for surgical planning, real-time intraoperative guidance, and patient education in complex spinal tumor cases.	Advantages: preoperative planning; intraoperative reference; anatomical visualization; patient education. Limitations: cost/resource burden; preparation time; limited comparative evidence.	3D-printed models may aid preoperative planning, intraoperative guidance, and patient education in complex spinal tumors. Future studies should quantitatively assess effects on operative time, surgical decision-making, and clinical outcomes.
13	Chen et al., 2019 [20], China	To design and evaluate a novel navigation template suitable for posterior cervical screw placement surgery using 3D printing.	3D printing was used to create individualized navigation templates for posterior cervical screw placement.	Advantages: navigation template; screw accuracy; safety; patient-specific planning. Limitations: soft-tissue exposure; template fragility; technical constraints.	The novel 3D-printed navigation template demonstrated high accuracy and safety for atlantoaxial PS placement. Careful soft-tissue removal, stable template handling, and attention to template fragility and drill-bit size are recommended.
14	Mohar et al., 2022 [27], Slovenia	To report the accuracy of PS placement in patients with thoracic scoliosis using PSGs.	PSG and 3D models of the deformed thoracic spine were created to guide the planning of PS placement.	Advantages: preoperative planning; screw trajectory accuracy; workflow efficiency; reduced surgical stress. Limitations: learning curve; selection bias; limited comparative assessment.	Patient-specific 3D templates may improve the safety and accuracy of thoracic PS placement in scoliosis. Future studies should evaluate comparative accuracy, efficiency, cost-effectiveness, radiation exposure, and the learning curve associated with this technique.
15	Xu et al., 2017 [36], China	To explore the application of 3D printing in assisting preoperative planning of PS placement for middle-upper thoracic trauma.	CT data were processed using Mimics software to create 1:1 3D fracture models for multiangle visualization and PS planning.	Advantages: preoperative planning; anatomical visualization; screw accuracy; accessibility/cost efficiency. Limitations: material constraints; outsourcing/privacy concerns; limited comparative evidence.	3D printing may assist PS fixation planning for middle-upper thoracic trauma by improving visualization and reducing empiric screw-placement errors. Early surgical planning and stabilization remain important for selected thoracic trauma cases.
16	Clifton et al., 2019 [21], USA	To determine the efficacy of a simulator for teaching neurosurgical residents the freehand technique of C2 laminar screw placement.	3D-printed C2 vertebrae models were created using multimaterial printing to simulate the corticocancellous bone interface for trainee practice.	Advantages: education/training; low-cost simulation; skill acquisition. Limitations: limited anatomical realism; narrow procedural scope.	The phantom prototype provided a low-cost simulator for neurosurgical trainees to practice freehand C2 laminar screw placement. Further refinement is needed to better simulate the in vivo environment.
17	Patchana et al., 2022 [28], USA	To describe the utility of 3D spine models in the training of thoracic PS placement.	3D-printed thoracic spine models were used in a hands-on training session to teach thoracic PS placement, allowing trainees to practice entry points and screw trajectories.	Advantages: education/training; screw trajectory practice; preoperative planning; cost efficiency. Limitations: limited intraoperative realism; limited study diversity.	3D-printed thoracic spine models may provide an affordable and effective training tool for thoracic PS placement. Further multi-institutional studies are needed to evaluate reproducibility and educational impact.
18	Braun et al., 2021 [18], Germany	To assess training of CT-guided peri-radicular therapy in a realistic simulation environment and derive recommendations for a training curriculum.	3D-printed patient-mimicking phantoms were used to train medical students to perform CT-guided periradicular therapy of the lumbar spine.	Advantages: education/training; realistic procedural simulation; skill acquisition. Limitations: limited procedural scope.	Simulation training for CT-guided periradicular therapy allows trainees to acquire fundamental skills in a safe environment and may improve confidence before supervised patient procedures.
19	Li et al., 2017 [26], China	To evaluate the accuracy and effect of a drill guide template for PS placement in severe scoliosis.	3D CT data were used to design drill guide templates for precise PS placement in severe scoliosis.	Advantages: navigation template; screw accuracy; reduced fluoroscopy; reduced operative burden. Limitations: cost/learning curve of navigation alternatives; need for technical planning.	The rapid-prototype pedicle drill template offered a precise and user-friendly approach for scoliosis surgery, with the potential to improve efficiency, safety, and radiation exposure. Knowledge of malformations remains essential for surgical planning and execution.
20	Xin et al., 2023 [34], China	To present a 3D osteotomy guidance system for thoracolumbar kyphosis osteotomy.	Preoperative planning and 3D-printed guide templates were used for thoracolumbar kyphosis osteotomy.	Advantages: preoperative planning; osteotomy guidance; intraoperative guidance; adaptability to deformity. Limitations: limited long-term comparative evidence.	The 3D osteotomy guidance system may bridge preoperative planning and intraoperative guidance in thoracolumbar kyphosis osteotomy. Long-term follow-up and comparison with freehand techniques are recommended.

Patient Demographics, Clinical Characteristics, Management, and Outcomes in the Non-comparative Retrospective Studies

Table 2 summarizes the demographic and perioperative characteristics of the 20 non-comparative retrospective studies [18-37]. Study size ranged from seven patients in Xu et al. [36] to 129 patients in Thayaparan et al. [33]. Reported mean or median ages varied across studies, and several deformity-focused studies included adolescent or mixed-age cohorts. Imaging modalities included CT in 16 studies [18,20,22-28,30-34,36,37], MRI in six studies [22-24,34,35,37], and X-ray in eight studies [20,22,23,27,30,31,34,36]. The included studies addressed cervical, thoracic, and lumbar pathologies, including scoliosis/kyphoscoliosis, degenerative disease, trauma, craniovertebral junction abnormalities, tumors, and ossification-related pathology. Management varied by study and included microsurgical decompression, minimally invasive transforaminal lumbar interbody fusion, screw fixation, navigation template-assisted procedures, osteotomy, and educational or simulation-based interventions. Because several studies were educational, simulation-based, or mixed-age deformity studies, demographic and outcome data were summarized descriptively and were not pooled quantitatively.

**Table 2:** Patient demographics, diagnostics, management, and outcomes in the non-comparative retrospective studies. 3D: three-dimensional; AIS: adolescent idiopathic scoliosis; AS: ankylosing spondylitis; ATPS: anterior transpedicular screw fixation; CT: computed tomography; CVJ: craniovertebral junction; F: female; LMS: lateral mass screw; PS: pedicle screw; M: male; MIS-TLIF: minimally invasive transforaminal lumbar interbody fusion; MRI: magnetic resonance imaging; PSO: pedicle subtraction osteotomy; TOLF: thoracic ossification of the ligamentum flavum; VAS: Visual Analog Scale; JOA: Japanese Orthopaedic Association; ASIA: American Spinal Injury Association; ODI: Oswestry Disability Index

#	Author, year, country	Study aim	Patient sex; patient age; medical history; presenting symptoms	Diagnostic imaging; pathology described; location	Management and outcomes: surgical management: average length of surgery (min), complications; conservative management, if any	Follow-up period (months); neurological outcome at last follow-up
1	Xin et al., 2022 [35], China	To develop a 3D-printed PSO guide plate system for patients with kyphosis.	4M, 6F; mean age: 51.9 years; kyphosis (10).	MRI (10); trauma (3), AS (2), degenerative changes (3), congenital deformities (2); T11 (1), T12 (2), L1 (5), L2 (1), L3 (1).	Osteotomy (10).	N/A
2	Ramirez et al., 2022 [29], Russia	To introduce a global, affordable 3D neuroanatomy teaching model for hands-on education.	N/A; educational model study without a clinical patient cohort.	N/A; neuroanatomy and spine-related educational modeling.	N/A; educational/simulation application only.	N/A
3	Burkhard et al., 2019 [19], Switzerland	To assess 3D-printed vertebrae for realistic haptic simulation of PS placement and decompression surgery.	N/A; simulation study without a clinical patient cohort.	3D-printed vertebral models; L3 vertebrae; surgical simulation of PS placement and decompression.	Posterior PS placement and decompression simulation; no clinical surgery.	N/A
4	Stefan et al., 2020 [31], Germany	To introduce a cost-effective 3D printing method for spine surgery simulation using CT-based synthetic bone models.	N/A; simulation study without a clinical patient cohort.	CT and X-ray-based synthetic spine bone models; spine surgery simulation.	Spine surgery simulation using 3D-printed CT-based synthetic bone models; no clinical surgery.	N/A
5	Govsa et al., 2018 [23], Turkey	To determine entry points for C1 LMS using 3D patient-specific spine models.	5M, 5F; age range: 15-35 years; C1 fractures.	CT, MRI, X-ray (10); C1 fractures (10); C1.	Screw fixation (10); no conservative management reported.	At least 1 month; significant reduction in arm neuralgia and arm pain.
6	Thayaparan et al., 2020 [33], Australia	To share the experience of using 3D-printed BioModels in 129 consecutive MIS-TLIF cases over 27 months, performed by one surgeon at a single center.	54M, 75F; mean age: 59.2 ± 13.1 years.	CT (129); disc disease (72), foraminal stenosis (65), canal stenosis (54), facet arthropathy (51), spondylolisthesis (47), scoliosis (13); lumbar.	MIS-TLIF surgery (129): 153.0 ± 43.8 min, range 55-298 min; dural tear (1), refractory hypotension (1), readmission for revision of implant position (4).	N/A
7	Zhao et al., 2017 [37], China	To evaluate clinical outcomes in microsurgical decompression of TOLF via a paraspinal approach using a percutaneous tubular retractor system.	7M, 6F; mean age: 53.2 years; lower limb weakness, numbness, and hypesthesia below T9 (4), urinary incontinence (7), gait disorder (1), progressive paraparesis and gait disorder (1), low back pain (1), post-trauma urinary incontinence, numbness, and neurological claudication (1), numbness and hypesthesia below T12 (2).	CT, MRI (13); TOLF; T9-11 (2), T10-11 (4), T9-10 (2), T7-10 (1), T6-7 (1), T8-10 (1), T10-12 (1).	Microsurgical decompression of TOLF via a paraspinal approach (13): 98.23 ± 19.10 min, range 80-134 min; no complications reported.	Average: 13.3 months, median: 12 months; preoperative JOA score: 4.15 ± 1.67; 1-month postoperative: 6.15 ± 0.69; 3 months: 6.38 ± 0.96; 6 months: 6.85 ± 0.90; final follow-up mean JOA score: 7.54 ± 1.13; recovery rate: 49.10 ± 15.71%; significant improvement observed (p < 0.05).
8	Li et al., 2018 [25], China	To create personalized navigation templates for insertion of lower cervical ATPS using 3D printing.	10M, 10F; mean age: 50.29 ± 6.98 years.	CT (20); lower cervical spine.	Insertion of lower cervical anterior transpedicular screws using personalized navigation templates (20).	N/A
9	Senkoylu et al., 2020 [30], Turkey	To evaluate the safety and effectiveness of patient-specific 3D rapid-prototype printing for PS insertion in AIS.	2M, 9F; mean age: 15 ± 1.8 years; AIS.	CT, X-ray (11); AIS (11); thoracic spine.	PS fixation using patient-specific 3D-printed guides (11); no complications reported.	N/A
10	Gao et al., 2017 [22], China	To evaluate the feasibility and efficacy of individualized 3D-printed model-assisted posterior internal fixation in treating CVJ abnormalities.	9M, 35F; mean age: 36.5 ± 9.2 years; atlantoaxial dislocation (41), Arnold-Chiari malformation (20), atlanto-occipital assimilation (39), basilar invagination (33), syringomyelia (12), platybasia (5), incomplete cervical segmentation (10); craniovertebral pain (29), incomplete paralysis (34), hemiplegia (4), gait disturbance (18), numbness/weakness in bilateral upper limbs (11), numbness/weakness in unilateral upper limb (5), expiratory dyspnea (4), trachyphonia with dysphagia (2), torticollis (4), diplopia (1), thenar muscle atrophy (6).	CT, MRI, X-ray (44); CVJ abnormalities (44); occiput-C2 (34), occiput-C2-C3 (6), C1-2 (4).	Posterior internal fixation (44); inspiratory dyspnea 0.5-1 hour after anesthesia recovery and tracheal cannula removal requiring immediate tracheotomy (1).	Average: 26 months, range 3-52 months.
11	Sugawara et al., 2017 [32], Japan	To evaluate the efficacy of methods for insertion of C1 LMS, C2 PS, and C2 laminar screws.	6M, 6F; mean age: 59.5 years, age range: 42-77 years; atlantoaxial instability (12), atlantoaxial dislocation (11), os odontoideum (1).	CT (12); atlantoaxial dislocation (11), os odontoideum (1); cervical spine.	Posterior C1-C2 fixation using patient-specific screw guide templates; C1 LMS, C2 PS, and/or C2 laminar screws placed according to planned trajectories.	N/A
12	Leary et al., 2021 [24], USA	To utilize 3D-printed models for preoperative planning and intraoperative guidance in complex spine tumors.	6M, 3F; mean age: 50.7 years.	CT, MRI (9); spinal tumors: chordoma (4), schwannoma (2), osteosarcoma (1), chondrosarcoma (1), Ewing-like sarcoma (1); cervical (3), cervicothoracic (1), thoracic (1), sacral (4).	Complex spinal tumor surgery with 3D-printed models used for preoperative planning, patient education, and intraoperative guidance; complex wound reconstruction reported in included cases.	N/A
13	Chen et al., 2019 [20], China	To design and evaluate a novel navigation template suitable for posterior cervical screw placement surgery using 3D printing.	7M, 5F; mean age: 45.5 years, range: 28-63 years.	CT, X-ray (12); atlantoaxial pathology with atlantodental interval ≤ 5 mm or space available for the cord ≤ 13 mm; cervical spine.	Atlantoaxial screw placement using individualized 3D-printed navigation templates (12): 37.67 ± 6.83 min total template-assisted placement time, 3.68 ± 0.99 min/piece; screw-placement-related complications such as nerve or vertebral artery injury were assessed.	N/A
14	Mohar et al., 2022 [27], Slovenia	To report the accuracy of PS placement in patients with thoracic scoliosis using patient-specific guides.	11M, 28F; mean age: 22.1 ± 13.7 years, range: 12.8-68.1 years.	CT, X-ray (39); thoracic scoliosis (39): idiopathic (31), congenital (1), neuromuscular (2), syndromic (1), adult revision deformities (4); thoracic spine.	Correction and posterior instrumented fusion (39): 348 ± 90 min, range 215-690 min; no complications reported.	Average: 66 months, range 42-102 months; neurological outcome not reported.
15	Xu et al., 2017 [36], China	To explore the application of 3D printing in assisting preoperative planning of PS placement for treating middle-upper thoracic trauma.	5M, 2F; mean age: 37.7 years; hemorrhage (4), renal/liver bruise (4), hemopneumothorax (2), pelvic fracture (2), craniofacial/skull bone fractures (5).	CT, X-ray (7); middle/upper thoracic trauma (7); T3-7 (2), T3-6 (1), T1-4 (1), T2-5 (1), T5-10 (1), T5-9 (1).	Spinal trauma procedure (7): 120 ± 30 min; replacement of Kirschner wire (2); conservative management: 3 stable fracture patients and 1 osteoporotic vertebral compression fracture patient received conservative treatment, details not specified.	More than 6 months; no issues with internal fixation observed on follow-up X-rays; 1 patient showed significant neurological improvement, 1 had mild improvement, and 5 had no significant change.
16	Clifton et al., 2019 [21], USA	To determine the efficacy of a simulator for teaching neurosurgical residents the freehand technique of C2 laminar screw placement.	N = 10 trainees; no clinical patient cohort.	3D-printed C2 vertebrae simulator; C1-C2 region; freehand C2 laminar screw placement training.	Simulation of freehand C2 laminar screw insertion; no clinical surgery.	N/A
17	Patchana et al., 2022 [28], USA	To describe the utility of 3D spine models in the training of thoracic PS placement.	N/A; educational/training study without a clinical patient cohort.	3D-printed thoracic spine models; thoracic PS placement training.	Hands-on thoracic PS placement simulation; no clinical surgery.	N/A
18	Braun et al., 2021 [18], Germany	To assess training of CT-guided periradicular therapy in a realistic simulation environment and derive recommendations for a training curriculum.	N/A; medical student training cohort; no clinical patient cohort.	CT-based lumbar spine phantom; CT-guided periradicular therapy simulation; lumbar spine.	CT-guided periradicular therapy simulation/training; no clinical surgery.	N/A
19	Li et al., 2017 [26], China	To evaluate the accuracy and effect of a drill guide template for PS placement in severe scoliosis.	5M, 3F; mean age: 16 years, range: 9-23 years; congenital scoliosis (5), idiopathic scoliosis (3).	CT (8); scoliosis (8); thoracic spine.	Vertebral osteotomy (5), simple internal fixation (3); no complications reported.	No spinal cord injury or nerve damage occurred; all patients had satisfactory outcomes.
20	Xin et al., 2023 [34], China	To present a 3D osteotomy guidance system for thoracolumbar kyphosis osteotomy.	4M, 3F; mean age: 52 years, range: 45-67 years; congenital kyphosis (1), traumatic kyphosis (6).	CT, MRI, X-ray (7); kyphosis (7); T10-L4 (1), T12 (1), L1 (2), L2 (1), L3 (2).	Osteotomy (7): 258 min, range 210-360 min; numbness in left lower limb resolving in 6 weeks (1).	Average: 7.4 months, range 4-12 months; no internal fixation displacement or fractures observed; ASIA improved from D to E in 6 cases and C to D in 1 case; VAS improved from 5.57, range 5-6, to 1.57, range 1-2; ODI improved from 40.71, range 36-45, to 10.71, range 9-13.

Utilization of 3D Printing in the Comparative Studies

Each article listed in Table 3 directly compared a 3D printing-assisted approach with a control or comparator approach within the same study [6-17]. The comparative studies reported outcomes including screw placement accuracy or acceptable screw placement, operative duration, estimated blood loss, fluoroscopy exposure, complications, follow-up duration, and patient-reported outcomes. Across these studies, several authors reported favorable findings associated with 3D printing or patient-specific guide use; however, outcome definitions, procedures, pathologies, and statistical reporting varied substantially. Therefore, comparative findings were summarized descriptively and were not pooled quantitatively. Patient-reported outcomes, including Visual Analog Scale (VAS), Oswestry Disability Index (ODI), and/or Neck Disability Index (NDI), were reported inconsistently across studies and are presented as study-level findings rather than review-level estimates of effect.

**Table 3:** 3D printing features, surgical and neurological outcomes, limitations, and conclusions in the comparative studies that assessed 3D printing for surgical pre-planning. 3D: three-dimensional; AO: atlanto-occipital; ASIA: American Spinal Injury Association; CSF: cerebrospinal fluid; CSM: cervical spondylotic myelopathy; CT: computed tomography; DVT: deep vein thrombosis; MRI: magnetic resonance imaging; ODI: Oswestry Disability Index; OPLL: ossification of posterior longitudinal ligament; PBG: positioning of bony gutter; AS: Visual Analog Scale; JOA: Japanese Orthopaedic Association; NDI: Neck Disability Index

#	Author, year. country	Study aim	Use of 3D	Compared groups	Complications	Acceptable rate (%); accuracy (%); mean angle of deviation (degrees)	Follow-up length and neurological outcome	Noted advantages or disadvantages of using 3D	Limitations noted in the study	Conclusions and recommendations
1	Kumagai et al., 2019 [9], Japan	To assess PBG precision in cervical laminoplasty with 3D-printed spine models.	CT scans were transformed into 3D models in 6-8 hours for surgical planning and anatomical visualization.	Group A: control; Group B: 3D-printed model	N/A	N/A	38.4 ± 8.1 months.	Advantage: precision in surgical planning with patient-specific anatomical models. Disadvantage: longer operation time observed in the 3D model group.	The study omitted X-ray assessment of cervical alignment, overlooked foraminal stenosis, included both CSM and OPLL patients, and lacked navigation technology.	3D-printed spine models may improve the precision of bony gutter placement in cervical laminoplasty by supporting patient-specific surgical planning.
2	Tan et al., 2018 [12], USA	To validate 3D-printed spine models for precise freehand pedicle screw placement in complex spinal deformity correction.	Intraoperative use of 3D-printed spine models facilitated identification of entry points and trajectories in dysmorphic or fused spines.	Group 1: 3D-printed model; Group 2: historical control	N/A	3D model: acceptable rate: 96.3%, accuracy: 84.2%, mean angle of deviation: N/A; control: N/A	N/A	Advantage: facilitates pedicle screw placement in complex spinal deformities. Disadvantage: none noted.	N/A	3D-printed spinal models may aid surgical planning and enhance safety during freehand pedicle screw insertion in complex spinal deformity by improving visualization of altered anatomy.
3	Aili et al., 2022 [6], China	To test preoperative planning with 3D-printed spine models for increased effectiveness and safety of spinal surgery.	Trajectories, screw lengths, and osteotomy details were designed using Mimics software and confirmed on 3D-printed spine models.	Group 1: 3D-printed model; Group 2: control	3D model: partial sensory impairment of left leg, spontaneously recovered (1); control: paraplegia nearly resolved within 1 year (1), CSF leakage (1)	3D model: accuracy: 87%, acceptable rate: N/A, mean angle of deviation: N/A; control: accuracy: 75%, acceptable rate: N/A, mean angle of deviation: N/A	Average follow-up: 16.2 ± 4.5 months, range 12-24 months. 3D model group: VAS decreased from 7.8 ± 2.3 to 1.5 ± 0.6; ODI decreased from 52.6 ± 15.3 to 28.5 ± 8.0. Control group: VAS decreased from 7.5 ± 1.9 preoperatively to 3.6 ± 1.3 at 4 weeks and 1.6 ± 0.7 at 1 year; ODI decreased from 50.8 ± 14.7 preoperatively to 38.8 ± 10.5 at 4 weeks and 26.7 ± 7.5 at 1 year.	Advantage: simplified preoperative planning and preparation for intraoperative challenges. Disadvantage: high cost and potential model inaccuracy.	Retrospective, single-center study with small sample size and possible selection bias.	3D-printed spine models may reduce operative time and blood loss, improve screw placement accuracy, and assist in deformity correction. Larger prospective multicenter studies are needed.
4	Zhuang et al., 2019 [17], China	To evaluate personalized 3D-printed models for enhancing patient comprehension of their medical condition and surgical plan.	3D printing was used for patient education in lumbar degenerative disease.	Group 1: CT and MRI imaging for patient education; Group 2: 3D reconstruction for patient education; Group 3: personalized 3D-printed model for patient education	N/A	N/A	N/A	Advantage: improved patient communication and education; potential cost-effectiveness as a teaching tool. Disadvantage: additional expense may limit widespread implementation.	The study focused on patient education rather than surgical outcome assessment.	Personalized 3D-printed models may improve patient understanding of lumbar degenerative disease and surgical planning discussions. Further validation is needed for effects on clinical outcomes.
5	Cecchinato et al., 2019 [7], Italy	To compare the accuracy of freehand pedicle screw insertion with 3D-printed patient-specific guides.	Patient-specific guide design was based on preoperative low-dose CT scans and 3D reconstruction of the spine.	Group A: pedicle screw implantation with MySpine patient-specific guides; Group B: freehand control	None reported in either group.	MySpine: accuracy: 90.2%, acceptable rate: N/A, mean angle of deviation: N/A; control: accuracy: 83.1%, acceptable rate: N/A, mean angle of deviation: N/A	N/A	Advantage: patient-specific guides reduced screw malpositioning and radiation exposure without increasing overall surgical duration. Disadvantage: none specifically reported.	Small sample size, open-label design, lack of blinding, reliance on low-dose CT for accuracy assessment, and limited follow-up.	Patient-specific guides decreased pedicle screw malpositioning, especially in complex spine surgery, while reducing radiation exposure and maintaining similar overall surgical duration.
6	Wang et al., 2021 [13], China	To assess 3D printing combined with guide plates for precise preoperative planning and secure pedicle screw placement in thoracic spinal tuberculosis surgery.	Computer virtual analysis guided virtual screw placement in a 3D-printed model, measuring screw dimensions and insertion details.	Group 1: 3D-printed guide plates for preoperative planning; Group 2: control	None reported in either group.	3D guide plate: accuracy: 98.86%, acceptable rate: N/A, mean angle of deviation: N/A; control: accuracy: 93.22%, acceptable rate: N/A, mean angle of deviation: N/A	Range: 6-12 months. 3D guide plate group: VAS 6 months 2.27 ± 1.04, VAS 12 months 1.53 ± 0.73, ODI 3 months 21.36 ± 6.50, ODI 6 months 10.67 ± 4.68. Control group: VAS 6 months 2.80 ± 0.92, VAS 12 months 2.03 ± 0.81, ODI 3 months 23.50 ± 6.52, ODI 6 months 13.07 ± 5.39.	Advantage: reduced surgical injury, shorter operative time, reduced X-ray radiation, and enhanced screw accuracy. Disadvantage: requires high-resolution imaging, time, specialized software, and equipment; limited utility in emergencies.	Study lacked pre-study mechanical testing and was limited by convenience sampling, subjective bias, and small sample size.	3D printing combined with guide plates may provide safe and effective pedicle screw placement for thoracic spinal tuberculosis. Further work should compare simulated versus actual screw placement and analyze operative time and blood loss.
7	Öztürk et al., 2022 [11], Turkey	To compare 3D model-assisted and conventional surgery for AO spine type-C injuries regarding surgery duration, fluoroscopy exposure, blood loss, and screw placement accuracy.	Patient-specific 3D models were used to study anatomy, simulate surgery preoperatively, and guide screw length, diameter, and approach angle selection.	Group 1: conventional surgery control; Group 2: 3D model-assisted surgery	N/A	Accuracy and acceptable rate were not consistently reported; mean angle of deviation was not reported.	N/A	Advantage: improved preoperative visualization, resident education, screw planning, and potential reduction in operative time, blood loss, and fluoroscopy exposure. Disadvantage: model preparation time limits emergency use.	Limited long-term clinical outcome assessment and limited evidence base for thoracolumbar type-C fracture surgery.	3D models helped surgeons visualize pathoanatomy and determine rod lengths, pedicle screw angles, and screw lengths preoperatively, potentially shortening operative time and reducing blood loss and fluoroscopy exposure.
8	Niu et al., 2022 [10], China	To compare freehand atlantoaxial pedicle screws with custom 3D-printed navigation template-assisted screws for upper cervical fractures.	A 1:1 3D-printed replica and individualized navigation template were developed before surgery.	Group A: custom 3D navigation template-assisted technique; Group B: freehand control	3D template: none; control: fever treated with antibiotics and dressing changes (1), CSF leakage resolving with intensive dressing changes (1)	3D template: accuracy: 95.7%, acceptable rate: N/A, mean angle of deviation: N/A; control: accuracy: 80.0%, acceptable rate: N/A, mean angle of deviation: N/A	N/A	Advantage: improved accuracy, reduced radiation exposure, and reduced learning curve difficulty compared with freehand technique. Disadvantage: computer-assisted screw insertion can be costly, complex, and sensitive to patient positioning.	Small sample size and retrospective design.	Patient-specific screw guide/template systems may improve C1-C2 screw navigation accuracy, reduce operation time, and minimize radiation exposure in an economical manner.
9	Kovalenko et al., 2021 [8], Russia	To compare individual navigation templates with intraoperative fluoroscopy for pedicle screw placement in the lumbar spine.	Individual 3D-printed navigation templates were used for subcortical pedicle screw placement in the lumbar spine.	Group 1: 3D-printed individual navigation templates; Group 2: intraoperative fluoroscopy control	Reoperation and incorrect implantation were described; number not specified. No neural structure damage was recorded in either group.	N/A	No damage to neural structures was recorded in either group.	Advantage: individual navigation templates may reduce implantation time and radiation exposure and improve screw placement safety. Disadvantage: lumbar template use may be limited by paravertebral muscles interfering with template positioning.	N/A	Individual navigation templates may enable safe pedicle screw implantation in the lumbar spine and reduce radiation exposure compared with fluoroscopy-guided implantation.
10	Ye et al., 2022 [16], China	To observe the clinical effect of posterior percutaneous long-segment internal fixation in ankylosing spondylitis patients with thoracolumbar fractures.	3D printing was used for precise preoperative planning and accurate placement of pedicle screws and rods.	Group 1: percutaneous long-segment internal fixation; Group 2: open fixation with long-segment screws.	Percutaneous group: high intraoperative blood loss (1), peri-implant infection (1); open group: DVT of lower extremities (2), postoperative anemia (2), bronchitis (1), intraoperative fracture displacement (1).	Cobb angle: percutaneous group 26.25 ± 12.64, open group 25.77 ± 12.35; accuracy/acceptable rate: N/A; mean angle of deviation: N/A.	Neurological improvement varied postoperatively; local decompression was performed for >4 mm bone block invasion or preoperative ASIA score indicating need.	Advantage: percutaneous fixation was associated with shorter operative time, less blood loss, less trauma, and faster recovery compared with open fixation. Disadvantage: none specifically reported for 3D printing.	Limitations included high cost, technical complexity, learning curve, fixed patient position, small sample size, retrospective design, and limited comparison because of insufficient data.	Minimally invasive posterior percutaneous long-segment fixation may reduce operative time, blood loss, and trauma in thoracolumbar fractures in ankylosing spondylitis. Larger comparative studies are needed.
11	Yang et al., 2018 [15], China	To assess the efficacy and feasibility of 3D-printed model-assisted posterior internal fixation.	A 1:1 scale 3D model was used to help diagnose fracture type, create preoperative plans, and select screw entry point and direction.	Group A: freehand control; Group B: 3D-printed model-assisted internal fixation	Control: migraine (6), CSF leakage (10), cerebral infarction (1), quadriplegia with death 1 month postoperatively (1); 3D model: migraine (4), CSF leakage (6)	N/A	Control: average: 42.7 months, range: 12-48 months; 3D model: average: 46.1 months, range: 12-46 months. As reported by the original study, both groups demonstrated postoperative improvements in VAS, JOA, and NDI at 1 week, 3 months, 6 months, and 12 months, with no significant intergroup differences.	Advantage: affordable 3D models, lower cost than navigation, improved screw placement, reduced time and blood loss. Disadvantage: time-intensive process, requiring 4-10 hours for CT data and model production; not suitable for urgent surgery; careful muscle removal required.	N/A	Compared with freehand technique, 3D-printed model-assisted posterior internal fixation may improve screw placement and reduce operative time and blood loss while providing an affordable guidance option.
12	Wu et al., 2023 [14], China	To compare the safety of 3D-printed flexible drill guiding templates with traditional rigid drill guiding templates for lower cervical pedicle screw insertion.	3D-printed flexible drill guiding templates were used for lower cervical pedicle screw fixation.	Group 1: 3D-printed flexible drill guiding templates; Group 2: 3D-printed regular/rigid drill guiding templates.	N/A	Deviation of screw entry point: flexible 0.65 ± 0.50 mm, rigid 0.78 ± 0.83 mm. Deviation of screw medial angle: flexible 2.14 ± 1.78°, rigid 4.23 ± 2.51°. Accuracy/acceptable rate not consistently reported in extracted table.	Average follow-up: 17.3 months, range 12-25 months; no significant difference in NDI between groups at 3 months postoperatively.	Advantage: flexible drill guide template reduced incision length, blood loss, and medial angle deviation and improved lower cervical pedicle screw safety. Disadvantage: none specifically reported.	Exclusion of lower cervical deformities, limited follow-up, and need for larger multicenter studies with long-term data.	Compared with regular guiding templates, lower cervical pedicle screw placement assisted by multistep navigation templates and flexible K-wires may result in less trauma and better safety.

Across the comparative studies, 3D printing was used primarily for preoperative anatomical visualization, patient-specific model-assisted planning, patient education, screw trajectory planning, and navigation template or drill guide development. Several studies reported favorable study-level findings associated with 3D printing or patient-specific guides, including improved screw placement accuracy or acceptable placement, shorter operative duration, reduced estimated blood loss, reduced fluoroscopy exposure, or fewer complications. However, these outcomes were reported inconsistently, and the studies differed substantially in pathology, procedure type, comparator group, 3D printing application, and outcome definitions. Therefore, these findings were summarized descriptively and were not pooled quantitatively. Reported limitations included upfront production cost, preparation time, imaging or radiation requirements, specialized software or equipment needs, template fit concerns, and limited generalizability.

Patient Demographics, Clinical Characteristics, Management, and Outcomes in the Comparative Studies

Table 4 summarizes the demographic, diagnostic, and perioperative characteristics of the 12 comparative studies [6-17]. These studies included heterogeneous populations with spinal deformity, degenerative disease, trauma, fracture or instability, cervical pathology, thoracic infection, and thoracolumbar pathology. Imaging most commonly included CT, often combined with MRI or radiographs depending on the indication. Surgical management varied substantially across studies and included decompression, screw fixation, laminoplasty, fusion, navigation template-assisted screw placement, percutaneous fixation, and open fixation. Because the comparative studies differed in pathology, surgical approach, comparator group, and reported outcome definitions, demographic and perioperative findings were summarized descriptively rather than pooled quantitatively.

**Table 4:** Patient demographics, diagnostics, management, and outcomes in the comparative studies. EBL: estimated blood loss; 3D: three-dimensional; AIS: adolescent idiopathic scoliosis; ADS: adult degenerative scoliosis; AO: atlanto-occipital; AS: ankylosing spondylitis; BMI: body mass index; CT: computed tomography; ed: education; F: female; PS: pedicle screw; M: male; MRI: magnetic resonance imaging; MVA: motor vehicle accident; OPLL: ossification of posterior longitudinal ligament; PBG: positioning of bony gutter

#	Author, year, country	Study aim	Compared groups	Patient sex; patient age; medical history; presenting symptoms		Diagnostic imaging; pathology described; location	Management		
							Conservative management	Surgical management	Average length (min); EBL (mL)
1	Kumagai et al., 2019 [9], Japan	Assess PBG precision in cervical laminoplasty with 3D-printed spine models.	Group A: control	12M, 3F; mean age: 58.5 ± 17.4 years		CT, MRI; cervical spondylotic myelopathy (6), OPLL (9); cervical, C3	N/A	C4-7 or C4-6 laminoplasty with a C3 laminectomy	174.1 ± 36.4 min; 158.7 ± 190.4 mL
			Group B: 3D-printed model	13M, 7F; mean age: 62.7 ± 11.5 years; Group B had significantly higher BMI (p = 0.030)					
2	Tan et al., 2018 [12], USA	Validate 3D-printed spine models for precise freehand PS placement in complex spinal deformity correction.	Group 1: 3D-printed model	10M, 13F; mean age: 35.7 years, age range: 16-67 years		Severe kyphoscoliosis, revision ADS, AS, hemi-vertebra, and distal junctional instrumentation failure; T1 to S1	N/A	PS placement	569.8 min; 1,753 mL
			Group 2: control (historical cohort)	3M, 17F		N/A			
3	Aili et al., 2022 [6], China	To test preoperative planning with 3D-printed spine models for increased effectiveness and safety of spinal surgery.	Group 1: 3D-printed model	13M, 15F; mean age: 25.6 ± 9.2 years, age range: 15-54 years		CT; congenital scoliosis (13), neuromuscular type scoliosis (7), idiopathic scoliosis (8); thoracolumbar (12), lumbar (12), upper thoracic (4)	Mentioned; use not specified	Internal fixation and spinal fusion using posterior surgical incision	375 ± 80 min; 363 ± 75 mL
			Group 2: control	10M, 15F; mean age: 29.1 ± 12.4 years, age range: 13-59 years		CT; congenital scoliosis (11), neuromuscular type scoliosis (6), idiopathic scoliosis (8); thoracolumbar (11), lumbar (10), upper thoracic (4)			456 ± 107 min; 442 ± 85 mL
4	Zhuang et al., 2019 [17], China	Evaluates personalized 3D-printed models for enhancing patient comprehension of their medical condition and surgical plan.	Group 1: CT & MRI imaging for patient ed	10M, 5F; mean age: 55 years		CT, MRI; lumbar degenerative disease (13), lumbar spinal stenosis (2); lumbar	N/A	Lumbar surgery	N/A
			Group 2: 3D reconstruction for patient ed	8M, 7F; mean age: 55 years		CT, MRI; lumbar degenerative disease (12), lumbar spinal stenosis (2), spondylolisthesis (1); lumbar			N/A
			Group 3: personalized 3D-printed model for patient ed	10M, 5F; mean age: 57 years		CT, MRI; lumbar degenerative disease (13), lumbar spinal stenosis (1), spondylolisthesis (1); lumbar			N/A
5	Cecchinato et al., 2019 [7], Italy	Comparing the accuracy of free-hand PS insertion to 3D-printed patient-specific guides.	Group A: PS implantation with MySpine™	2M, 12F; mean age: 34 ± 15.3 years		CT, X-ray; AIS (6), ADS (6), congenital deformities (2)	N/A	PS instrumentation and posterior-only or combined approaches in the same session	7 hrs, 2 min, and 26 sec
			Group B: control	1M, 14F; mean age: 26 ± 17.2		X-ray; AIS (10), ADS (4), congenital deformities (1)	N/A		7 hrs, 3 min, and 51 sec
6	Wang et al., 2021 [13], China	Assess 3D printing with guide plates for precise preoperative planning and secure PS placement in thoracic tuberculosis surgery.	Group 1: 3D-printed guide plates for preoperative planning	16M, 14F; age range: 42-51 years; N/A; chest and back pain with or without spinal cord compression symptoms		CT, MRI, X-ray; thoracic spine tuberculosis; T1-2 (1), T2-3 (1), T3-4 (1), T4-5 (3), T5-6 (3), T6-7 (5), T7-8 (6), T8-9 (2), T9-10 (2), T10-11 (3), T11-12 (3)	Regular antituberculosis preoperative treatment for >3 weeks	Thoracic spinal tuberculosis surgery with nail placement	137.67 ± 9.39 min; 599.33 ± 83.37 mL
			Group 2: control	15M, 15F; age range: 41-52 years; N/A; chest and back pain with or without spinal cord compression symptoms		CT, MRI, X-ray; thoracic spine tuberculosis; T1-2(1), T2-3 (1), T3-4 (1), T4-5 (2), T5-6 (2), T6-7 (6), T7-8 (7), T8-9 (2), T9-10 (1), T10-11 (4), T11-12 (3)			170.00 ± 20.48 min; 674.6 ± 83.61 mL
7	Öztürk et al., 2022 [11], Turkey	To compare 3D model-assisted and conventional surgery for AO spinal C-type injuries regarding surgery duration, fluoroscopy exposure, blood loss, and screw placement accuracy.	Group 1: control	8M, 8F; mean age: 38.8 ± 13.4 years; injury due to: fall (9), traffic accident (7); associated trauma: head injury (3), pulmonary symptoms (4), extremity fracture (3), pelvic fracture (2), abdominal trauma (2)		CT; thoracolumbar, AO spinal C-type injuries; T1-10 (3), T11-L2 (13)	N/A	Long-segment posterior instrumentation	75.5 ± 11.0 min; 347.8 ± 52.2 mL
			Group 2: 3D model-assisted surgery	6M, 10F; mean age: 37.2 ± 13.8 years; injury due to: fall (13), traffic accident (3); associated trauma: head injury (2), pulmonary symptoms (5), extremity fracture (2), pelvic fracture (3), abdominal trauma (1)		CT; thoracolumbar, AO spinal C-type injuries; T1-10 (4), T11-L2 (12)			61.9 ± 4.7 min; 268.4 ± 42.7 mL
8	Niu et al., 2022 [10], China	Compares the efficacy and safety of freehand atlantoaxial PS against custom 3D-printed navigation template screws in treating upper cervical fractures.	Group A: custom 3D-printed navigation template-assisted screw technique	9M, 3F; mean age: 47.8 ± 11.4 years; upper cervical fractures due to traffic accident (6), fall from height (3), fall on the ground (3); neck pain (12), dysphagia (5), paresthesia/weakness (9)		CT, X-ray; Upper cervical fractures; Cervical	N/A	Screw fixation	110.0 ± 31.9 min; 159.6 ± 90.1 mL
			Group B: control	9M, 2F; mean age: 42.5 ± 16.7 years; upper cervical fractures due to traffic accident (5), fall from height (1), fall on the ground (5); neck pain (11), dysphagia (4), paresthesia/weakness (8)		CT, X-ray; upper cervical fractures; cervical			173.8 ± 53.3 min; 304.0 ± 167.5 mL
9	Kovalenko et al., 2021 [8], Russia	Assess PS implantation in lumbar spine: navigation templates vs. intraoperative fluoroscopy for efficacy comparison.	Group 1: 3D-printed navigation templates were used	N/A; mean age: 54 years, range: 37-71 years		CT, X-ray; compression; lumbosacral	N/A	Decompression and stabilization with screw fixation	119.0 min (range: 108.0-128.75 min)
			Group 2: control			CT, X-ray; compression; lumbosacral			173.0 min (range: 155.0-192.25 min)
10	Ye et al., 2022 [16], China	Observe the clinical effect of posterior percutaneous long-segment internal fixation in 26 AS patients with thoracolumbar fractures.	Group 1: percutaneous long-segment internal fixation (n = 26)	36M, 11F	Mean age: 58.12 ± 15.72 years, age range: 29-85 years	CT, MRI; AS; thoracolumbar	N/A	Percutaneous long‑segment internal fixation or open fixation with long‑segment screws	127.27 ± 21.17 min; 292.15 ± 177.66 mL
			Group 2: open fixation with long-segment screws (n = 21)		Mean age: 55.43 ± 14.83 years, age range: 35-86 years				153.29 ± 78.10 min; 409.81 ± 101.47 mL
11	Yang et al., 2018 [15], China	Assess the efficacy and feasibility of 3D printing models-assisted posterior internal fixation.	Group A: control	36M, 40F; mean age: 51.3 ± 7.4 years, age range: 39-61 years; rheumatoid arthritis (4); atlantoaxial instability due to rheumatoid arthritis (4), other causes of atlantoaxial instability (17)		CT, MRI, X-ray; atlantoaxial fracture and dislocation (51), congenital odontoid nonunion (7); cervical	N/A	C1 PS fixation, inserted using the Resnick method	159.4 ± 15.6 min; 164.6 ± 28.4 mL
			Group B: internal fixation assisted by 3D printing	26M, 36F; mean age: 49.8 ± 6.6 years, age range: 42-59 years; rheumatoid arthritis (6); atlantoaxial instability due to rheumatoid arthritis (6), other causes of atlantoaxial instability (8)		CT, MRI, X-ray; atlantoaxial fracture and dislocation (48), congenital odontoid non-union (5); cervical			105.7 ± 14.6 min; 114.3 ± 14.6 mL
12	Wu et al., 2023 [14], China	This research examined the safety of 3D-printed flexible drill guiding templates in contrast to traditional rigid templates for the insertion of PS in the lower cervical spine.	Group 1: 3D-printed flexible drill guiding templates	12M, 6F; mean age: 37.9 ± 9.5 years; trauma: MVA (9), high energy fall (7), other (2); N/A		CTA; lower cervical fracture or instability diagnosed after trauma; C3-C7	N/A	Posterior cervical fixation	95.7 ± 15.4 min; 128.9 ± 30.5 mL
			Group 2: 3D-printed regular drill guiding templates	11M, 5F; mean age: 38.2 ± 6.1 years; trauma: MVA (8), high-energy fall (6), other (2); N/A					132.6 ± 26.5 min; 253 ± 50.7 mL

Utilization of 3D Printing in the Case Reports 

The 39 case reports and technical reports described the use of 3D printing for surgical planning and related spine applications (Table 5). Recurring themes included complex deformity, tumor resection or reconstruction, trauma, craniovertebral junction pathology, patient-specific screw or osteotomy guides, surgical rehearsal, intraoperative reference, patient education, and selected implant or prosthetic applications. Several reports described perceived benefits such as improved anatomical visualization, spatial orientation, operative planning, patient communication, or intraoperative decision-making. However, these reports were heterogeneous and generally did not provide controlled outcome data. Reported limitations included cost, production time, imaging requirements, limited soft-tissue representation, and uncertainty regarding generalizability.

**Table 5:** Case reports included in the review. 3D: three-dimensional

#	Title	First author, year	Study aim
1	Partial sacrectomy with patient-specific osteotomy guides [38]	Farshad et al., 2021	To present a case using 3D-printed guides for sacrococcygeal chordoma resection in a 49-year-old woman.
2	En bloc resection of primary malignant bone tumor in the cervical spine based on 3-dimensional printing technology [47]	Xiao et al., 2016	To assess the safety and utility of en bloc resection through a combined anterior and posterior approach for cervical primary malignant bone tumors using 3D preoperative 3D-printed models.
3	Application of 3D-printed model in the cervical spine osteochondroma surgery: a case report [48]	Liao et al., 2021	To present a case using a 3D-printed model to prepare for surgery through educating a patient and their family preoperatively about the upcoming surgical procedure and rehearse the procedure.
4	Virtual scoliosis surgery using a 3D-printed model based on biplanar radiographs [44]	Courvoisier et al., 2022	To describe a protocol for the surgical procedure correcting adolescent idiopathic scoliosis using 3D-printed spine models to simulate the surgery.
5	3D-printed titanium prosthetic reconstruction of unilateral bone deficiency after surgical resection of tumor lesions in the upper cervical spine: clinical outcomes of three consecutive cases and narrative review [49]	Shen et al., 2023	To describe the use of 3D printing in the reconstruction of unilateral bony deficits following resection of a giant cell tumor.
6	Multidisciplinary surgical planning for en bloc resection of malignant primary cervical spine tumors involving 3D-printed models and neoadjuvant therapies: report of 2 cases [50]	Ahmed et al., 2019	To present two cases of primary cervical spine tumors managed with en bloc resection and neoadjuvant therapies using 3D-printed patient models.
7	Designing patient-specific 3D printed devices for posterior atlantoaxial transarticular fixation surgery [45]	Thayaparan et al., 2018	To report the utility and effectiveness of biomodeling and 3D printing in posterior transarticular atlantoaxial fixation surgery for the development of patient-specific solutions.
8	The utility of 3D printing for surgical planning and patient-specific implant design for complex spinal pathologies: case report [46]	Mobbs et al., 2017	To evaluate the use of 3D printing for surgical planning and implant design for complex spinal surgery cases.
9	Applying 3-dimensional printing and modeling for preoperative reconstruction and instrumentation placement planning in complex deformity surgery [42]	Caruso et al., 2022	To demonstrate a cost-effective approach for the development of patient-specific 3D-printed spine models of severe spinal deformities to improve informed consent, trainee education, and planning.
10	3D printing model of a patient's specific lumbar vertebra [39]	Bai et al., 2023	To present the use of a 3D-printed patient-specific lumbar vertebra model for planning, surgical navigation, and training in selective dorsal rhizotomy.
11	Anterior lumbar interbody fusion using a personalized approach: is custom the future of implants for anterior lumbar interbody fusion surgery? [40]	Mobbs et al., 2019	To assess the utility and benefit of 3D printing in spinal surgery cases with complex anatomic disease.
12	Patient-specific processes for occipitocervical fixation using biomodelling and additive manufacturing [51]	Thayaparan et al., 2020	To present a case of occipitocervical fusion with a 3D-printed titanium implant in a 79-year-old female utilizing a 1:1 scale 3D-printed biomodel for planning, education, and intraoperative reference.
13	A modern multidisciplinary approach to a large cervicothoracic chordoma using staged en bloc resection with intraoperative image-guided navigation and 3D-printed modeling: illustrative case [43]	Pertsch et al., 2021	To present a case of successful en bloc resection using a 3D-printed patient-specific model for the management of a complex cervicothoracic junction chordoma.
14	Posterolateral epidural supra-C2-root approach (PESCA) for biopsy of lesions of the odontoid process in same sitting after occipitocervical fixation and decompression-perioperative management and how to avoid vertebral artery injury [52]	Haas et al., 2021	To present the posterolateral epidural supra-C2-root approach as an alternative to transoral, anterolateral, and other posterolateral approaches for biopsy of the odontoid process, using 3D-printed models for surgical planning.
15	Three-dimensional planning and patient-specific drill guides for repair of spondylolysis/L5 pars defect [53]	Mobbs et al., 2019	To present a case of a 28-year-old woman managed with compression screw placement using a patient-specific drill guide and 3D-printed vertebrae model.
16	Three-dimensional printing: an aid to epidural access for neuromodulation [54]	Taverner and Monagle, 2017	To demonstrate the utility of 3D printing in neuromodulation.
17	Case report: challenging post-traumatic pseudoarthrosis of C2 odontoid fracture and extreme C1-C2 subluxation [55]	Abreu et al., 2022	To demonstrate a case in which a 3D-printed model was constructed for surgical planning prior to a 1 posterior osteotomy.
18	Occipitocervical fusion combined with 3-dimensional navigation and 3-dimensional printing technology for the treatment of atlantoaxial dislocation with basilar invagination: a case report [56]	Yuan et al., 2020	To demonstrate a case using 3D printing to reduce the risk of surgical complications in the management of a basilar invagination.
19	Resection of giant invasive sacral schwannoma using image-based customized osteotomy tools [57]	Lin et al., 2016	To assess virtual surgical planning, computer-aided design, and manufacturing with 3D printing for the development of patient-specific osteotomy guides in the management of giant invasive sacral schwannoma.
20	Cervical myelopathy in a patient with Klippel-Feil syndrome treated with a patient-specific custom cervical spine locking plate [58]	Jackson et al,. 2022	To report a case of Klippel-Feil syndrome managed with anterior C5 corpectomy and arthrodesis in which a 3D-printed model was used to facilitate preoperative evaluation.
21	Cortical bone trajectory screw placement accuracy with a patient-matched 3-dimensional printed guide in lumbar spinal surgery: a clinical study [59]	Marengo et al., 2019	To evaluate the use of patient-specific 3D-printed target guides for fixation with cortical bone trajectory screws.
22	Design of mulitlevel OLF approach ("V"-shaped decompressive laminoplasty) based on 3D printing technology [60]	Ling et al., 2018	To present a new surgical approach using 3D-printed models in multilevel ossification of the ligamentum flavum.
23	Three-dimensional-printed individualized guiding templates for surgical correction of severe kyphoscoliosis secondary to ankylosing spondylitis: outcomes of 9 cases [61]	Tu et al., 2019	To present the outcome of using 3D-printed models and guiding templates in the planning and management of severe kyphoscoliosis secondary to ankylosing spondylitis.
24	Rapid personalised virtual planning and on-demand surgery for acute spinal trauma using 3D-printing, biomodelling and patient-specific implant manufacture [41]	Mobbs et al., 2022	To assess the utility of 3D printing in the management of emergency traumatic spinal injuries.
25	Preoperative planning using three-dimensional printing for full-endoscopic spine surgery: a technical note [62]	Okada et al., 2022	To improve the safety and efficacy of transforaminal full-endoscopic spine surgery through the use of 3D printing in preoperative planning.
26	No significant effect of 3D modelling on surgical planning in spinal deformities [63]	Guran et al., 2022	To assess the impact of 3D-printed models on surgical planning for the management of complex spinal deformities.
27	Bilateral pure facet joint dislocation in thoracolumbar junction (T11-T12) without facet fracture using a 3D digital printing model for surgical planning: a case report [64]	Liawrungrueang et al., 2020	To present a case of 3D printing used in preoperative planning and follow-up of an open reduction, decompression, and instrumentation with posterolateral fusion in a 25-year-old man.
28	Complicated postoperative flat back deformity correction with the aid of virtual and 3D printed anatomical models: case report [65]	Fayad et al., 2021	To present a case of a 71-year-old woman with a complex anatomical condition in which 3D printing was used to develop an appropriate surgical plan.
29	Biomechanical evaluation and the assisted 3D printed model in the patient-specific preoperative planning for thoracic spinal tuberculosis: a finite element analysis [66]	Wang et al., 2020	To analyze the application of assisted finite element analysis and 3D-printed models for preoperative planning in thoracic spinal tuberculosis.
30	A tailored approach to cortical bone track for spine fixation surgery: 3-dimensional printed custom made guides for screws placement: 2-dimensional operative video [67]	Marengo et al., 2020	To present a case of a 46-year-old male undergoing cortical bone trajectory screw fixation using 3D-printed patient-specific guides.
31	Surgical orthopedics in a spondylometaphyseal dysplastic patient [68]	Zhang et al., 2018	To demonstrate the feasibility and effectiveness of vertebral column resection for spondylometaphyseal dysplasia using 3D printing.
32	Pseudarthrosis repair using autologous cultured osteoblasts in complex type-1 neurofibromatosis spinal deformity: a case report and review of the literature [69]	Kim et al., 2016	To present a case of late vertebral dislocation in which 3D printing was used for understanding of surgical anatomy prior to pseudarthrosis repairs in a patient with progressive dural ectasia and type-1 neurofibromatosis.
33	Coordinated approach to spinal and tracheal reconstruction in a patient with Morquio syndrome [70]	Kiessling et al., 2020	To present a case of Morquio syndrome with cervical and upper thoracic spinal stenosis in which 3D-printed anatomical models were developed for preoperative planning.
34	Development of patient-specific phantoms for verification of stereotactic body radiation therapy planning in patients with metallic screw fixation [71]	Oh et al., 2017	To evaluate a new technique for manufacturing a patient-specific dosimetric phantom with 3D printing.
35	An innovative prone positioning system for advanced deformity and frailty in complex spine surgery [72]	Kolb et al., 2019	To assess a prone positioning system for cervical extension osteotomy in ankylosing spondylitis using a 3D model for simulation and verification.
36	Patient-centered oncosurgical planning with cancer models in subspecialty education [73]	Guler et al., 2021	To determine the impact of 3D-printed patient-specific models on the development of operative plans by resident-level trainees.
37	Development and first clinical use of a novel anatomical and biomechanical testing platform for scoliosis [74]	Bohl et al., 2019	To report the development of a 3D-printed biomimetic scoliosis model and its utility for surgical planning and education.
38	Accuracy of axis drill guides in the cases of atlantoaxial instabilities associated with high-riding vertebral arteries, narrow pedicles, and complex deformities: comparison of 3 fixation methods [75]	Malikov et al., 2022	To evaluate variation in the accuracy of pedicle and/or pars screw fixation using 3D-printed C2 navigation templates versus freehand techniques.
39	Clinical application of personalized digital surgical planning and precise execution for severe and complex adult spinal deformity correction utilizing 3D printing techniques [76]	Ding et al., 2023	To present the clinical application of 3D printing in personalized preoperative planning and as guidance templates for the management of severe and complex adult spinal deformity.

Among the additional reports summarized in Table 5, many described favorable practical utility or supported selected use of 3D printing for preoperative planning, intraoperative reference, patient-specific guide development, implant customization, education, or simulation. However, several reports emphasized the need for further validation before standardizing the widespread clinical use of 3D printing in spine surgery. Oh et al. [71] highlighted the importance of assessing the balance between potential benefits and risks, including radiation exposure related to imaging required for patient-specific model development. Guran et al. [63] reported no significant effect of 3D modeling on surgical planning in spinal deformity, underscoring that not all cases may benefit equally from 3D printing workflows. Overall, these reports support selected and case-specific use of 3D printing rather than routine use for all spine surgery planning.

Discussion

The applications of 3D printing in spine surgery extend beyond preoperative planning and include intraoperative reference, patient-specific guides, selected custom implants, patient communication, and trainee education. In the included literature, patient-specific models were most often used to improve anatomical visualization, support surgical rehearsal, assist trajectory planning, and facilitate communication among surgeons, trainees, and patients. However, the current evidence does not establish definitive improvement in long-term clinical outcomes. Rather, the available studies suggest that 3D printing may serve as a useful adjunct in selected complex cases, particularly when conventional two-dimensional or screen-based imaging does not fully convey patient-specific anatomy. While previous reviews have described broader clinical use cases or provided general commentary on 3D printing in spine surgery [77,78], the present scoping review maps applications across preoperative planning, education, simulation, intraoperative reference, patient-specific guides, and selected implant customization, while emphasizing the limitations of the current evidence base.

Preoperative Planning Using 3D Printing

Preoperative planning is one of the most widespread applications of 3D printing in spine surgery. Since 3D printing is a multistep process that involves image acquisition and software preprocessing before the actual printing, the quality and accuracy of the models printed are inextricably linked to the quality offered by the imaging modality [79]. Cecchinato et al. [7] demonstrated that employing low-dose CT scans, although it decreases radiation exposure by 80% per pedicle, only accurately represents the bony outline and not the internal core in printed reconstructions. The need for high-fidelity imaging might explain the prevalence of high-quality CT and MRI in many retrospective studies and individual patient cases included in our review.

Although 3D imaging techniques can generate virtual 3D models of the spine, they often lack the tactile feedback and tangible nature inherent in 3D-printed models [21,28]. This tangible aspect is particularly valuable in surgical planning and education, as it allows surgeons to physically manipulate and interact with the models, enhancing their understanding and spatial awareness [35]. However, software limitations, segmentation variability, and image-processing requirements can compromise the realism and precision of these models, particularly in complex anatomical cases. In addition, the need for high-resolution imaging, specialized software, technical expertise, printing equipment, and post-processing can limit accessibility and scalability, especially in lower-resource settings or institutions without dedicated 3D printing infrastructure [5,13]. Addressing these limitations will be crucial in ensuring equitable access to the benefits of 3D printing technology in spine and neurosurgery. Ramirez et al. [29] described a low-cost 3D modeling approach that may help reduce educational gaps between low- and high-resource training environments. By enabling the creation of highly realistic patient-specific and often life-size models, 3D printing allows for an authentic and realistic reflection of bony anatomy across deformities and spinal levels such as in Govsa et al. [23].

3D-printed replicas may be particularly useful in cases involving complex anatomy or pathology because the models can be physically held, rotated, and inspected from multiple angles without requiring the surgeon to mentally reconstruct 3D anatomy from two-dimensional images. This may explain why many case reports and technical reports involved complex deformity, degenerative disease, ankylosing spondylitis, trauma, tumor, or craniovertebral junction pathology. Moreover, precise 1:1 replicas may support practical preoperative measurements, including planned entry points, screw trajectories, osteotomy margins, implant sizing, and rod contouring [14,28,30,80]. When used intraoperatively, these models may also serve as references for confirming exposed bony landmarks, planned trajectories, or anticipated maneuvers after the relevant anatomy has been exposed [14,28,30,80].

Education and Training Using 3D Printing

Enhancements in 3D printing materials have elevated model quality to closely mimic native bones. Introduction of such properties makes these models more attractive for use in simulated learning environments, particularly where tactile and haptic stimulation is crucial for training and education. While historically, cadavers have been primarily employed for hands-on training, the associated costs are high, and the specimens pose an additional health risk. 3D model-based simulations not only enable more individuals to undergo training but also facilitate the customization of learning objectives. Several non-comparative studies included in this review used 3D models for education, simulation, or training purposes [18,19,21,28,29,31]. For example, Braun et al. [18] created a realistic simulation environment based on 3D-printed phantoms and were able to establish a learning curve for medical students performing CT-guided periradicular therapy. Such simulation studies may help generate recommendations for trainee preparation and curriculum development. Another application of 3D-printed models found in our review is their assistance in intraoperative screw guides. These templates aid surgeons in guiding screws, trocars, or drills to specific locations, although they often require validation on anatomical structures [7,10-14,16,26]. Conforming 3D-printed models to the surface of the guides allows surgeons to plan and practice approaches before the actual operation, enhancing precision and reducing intraoperative challenges. When applied to clinical practice, 3D printing workflows may help surgeons plan selected cases more effectively and may support education by providing repeatable, patient-specific, or procedure-specific simulation models. However, the clinical effect of these educational and planning applications remains incompletely defined, and future studies should distinguish educational benefit, technical accuracy, operative efficiency, and patient-centered outcomes using standardized measures.

Outcomes Associated With 3D Printing

The influence of 3D-printed models on procedural outcomes has been evaluated through direct technical measures, including screw placement accuracy or acceptable screw placement, and indirect perioperative measures such as operative duration, estimated blood loss, fluoroscopy exposure, complications, and patient-reported outcomes. Several comparative studies reported favorable study-level findings associated with 3D printing or patient-specific guides [6-17]. However, these outcomes were reported inconsistently across studies, and the included literature varied substantially with respect to study design, pathology, spinal region, procedure type, use of anatomical models versus templates, imaging assessment method, outcome definition, and statistical reporting. Therefore, quantitative pooling was not performed. These findings should be interpreted as descriptive, hypothesis-generating trends rather than pooled estimates of effect or definitive evidence of improved clinical outcomes.

Although several studies identified the upfront cost of model production, software, equipment, and personnel as limitations of 3D printing, some comparative studies suggested that these initial costs may be offset in selected contexts by downstream factors such as shorter operative duration, reduced blood loss, lower fluoroscopy exposure, improved implant planning, fewer complications, or shorter hospitalization. Therefore, cost should be interpreted as context-dependent: 3D printing may increase preoperative preparation costs while potentially reducing total episode-of-care costs in selected cases. However, formal cost-effectiveness data remain limited.

Limitations of Using 3D Printing

Despite the perioperative advantages reported in selected studies, several limitations remain associated with the use of 3D printing in spine care. The software platforms, segmentation methods, imaging protocols, printing materials, printer technologies, post-processing steps, sterilization requirements, and validation methods varied substantially across studies [21,24]. This lack of standardization limits reproducibility, complicates cross-study comparison, and may affect model accuracy, template fit, and clinical implementation. Available regulatory and terminology frameworks, including FDA technical considerations for additive-manufactured medical devices and International Organization for Standardization (ISO)/American Society for Testing and Materials (ASTM) additive manufacturing terminology standards, provide important guidance for device development and reporting; however, standardized spine-specific workflows for image acquisition, segmentation, model validation, intraoperative use, and outcome reporting remain incompletely defined [81,82]. As more institutions adopt 3D printing workflows, future studies should emphasize reproducible protocols, quality assurance, dimensional accuracy testing, cost reporting, and standardized clinical endpoints. Additionally, although 3D-printed models may assist with operative planning, the time required for model design and production ranged from a few hours to several days or weeks across included studies, limiting use in emergent surgical procedures [22,24,29,32].

Utility of 3D Printing in Practice

Despite limitations related to cost, preparation time, and technical requirements, the included studies suggest that 3D-printed models may be useful adjuncts for selected complex spine surgery cases [21,22,24,29,32]. Their tactile nature and ability to display patient-specific anatomy from multiple angles may improve spatial understanding, support rehearsal of planned trajectories or osteotomies, and help anticipate intraoperative challenges. However, these models should supplement rather than replace conventional imaging review, surgical judgment, and established planning workflows. In complex procedures, 3D-printed models may be most appropriately viewed as confirmatory and clarifying tools rather than standalone planning methods.

Limitations and Future Directions

This review acknowledges several limitations. Firstly, the reliance on predefined inclusion criteria may introduce selection bias. Additionally, the retrospective nature of several studies increases the risk of selection bias. Furthermore, publication bias may impact our findings, as studies with positive outcomes are more likely to be published. Moreover, our focus on 3D printing in perioperative planning and education may exclude studies exploring other applications of 3D printing in surgery. Another limitation is the exclusion of pediatric studies. This decision limits the applicability of our conclusions to pediatric spine surgery, where 3D printing may be particularly valuable for congenital scoliosis, spinal dysraphism, kyphoscoliosis, and other complex deformities. However, pediatric spine surgery differs substantially from adult spine surgery with respect to growth-related anatomy, surgical indications, implant strategy, and outcome assessment. As a result, pediatric applications of 3D printing warrant dedicated evaluation in future studies.

A major limitation of the current evidence is the lack of standardized outcome reporting across included studies. Terms such as accuracy rate, acceptable rate, and angle of deviation were not consistently defined, and studies varied in whether screw placement was assessed by postoperative CT, radiographs, cortical breach grading, millimeter deviation, surgeon judgment, or other criteria. In addition, outcomes were reported inconsistently at the screw, patient, or procedure level, making direct cross-study comparison unreliable. Similar variability was present for operative duration, estimated blood loss, fluoroscopy exposure, complications, follow-up duration, and patient-reported outcomes. As a result, the outcome data in this review should be interpreted as descriptive and hypothesis-generating rather than as quantitatively comparable estimates of effect. Future studies should use standardized definitions, validated grading systems, consistent reporting units, and uniform follow-up intervals to improve comparability across studies.

Additional methodological limitations should also be considered. A protocol for this scoping review was not prospectively registered, which limits external verification that all eligibility criteria, extraction variables, and synthesis decisions were finalized before data abstraction. In addition, articles not available in English were excluded, introducing potential language bias. Although a broad search strategy was used, variation in terminology across the 3D printing literature may have resulted in missed studies that used alternative terms not captured by the search strategy. Finally, consistent with the scoping review objective of mapping the available literature, a formal quality appraisal or risk-of-bias assessment was not performed. Therefore, the findings should be interpreted as a descriptive map of the literature rather than as a graded assessment of evidence certainty or comparative treatment effectiveness.

Future research should additionally investigate the broader utility of 3D printing, including its role in surgical guide development, implant customization, patient-specific instrumentation, and intraoperative applications. Emerging technologies such as intraoperative navigation, robotic guidance, augmented reality-assisted spine surgery, and 3D image-guided workflows represent complementary approaches to patient-specific preoperative planning. Although these technologies differ from physical 3D printing, they share the goal of improving spatial understanding, screw trajectory planning, and intraoperative precision, particularly in complex deformity, trauma, tumor, and craniovertebral junction cases. Future studies should also evaluate how 3D-printed models, navigation platforms, augmented reality, and robotic systems can be integrated into standardized planning workflows.

## Conclusions

3D printing appears to be a promising adjunct for selected spine surgery applications, particularly preoperative anatomical visualization, surgical rehearsal, patient and trainee education, screw trajectory planning, navigation template development, intraoperative reference, and selected patient-specific implant or prosthetic applications. However, the current evidence remains largely retrospective, heterogeneous, and descriptive, with many studies limited by small sample sizes, single-center designs, inconsistent outcome definitions, and limited long-term follow-up. The available literature supports the feasibility and potential utility of 3D printing in complex spine surgery workflows, but it does not establish definitive evidence of improved long-term clinical outcomes or justify routine use as a standard component of preoperative planning. Prospective, multicenter, controlled studies using standardized outcome measures, cost-effectiveness analyses, validated accuracy definitions, and longer follow-up are needed to determine where 3D printing provides the greatest clinical value in spine surgery.

## Appendices

Database search strategies

The final database searches were completed in September 2025. No date restrictions were applied. Articles not available in English were excluded during eligibility screening.

PubMed

Search strategy:

(("3D printing" OR "three-dimensional printing" OR "3D printed" OR "additive manufacturing" OR "rapid prototyping" OR stereolithography OR "patient-specific model*" OR "printed model*" OR "navigation template*" OR "patient-specific guide*") AND (spine OR spinal OR vertebra* OR scoliosis OR kyphosis OR "pedicle screw"))

Screening method: All retrieved titles and abstracts were screened.

Embase

Search strategy:

(‘3d printing’ OR ‘three dimensional printing’ OR ‘additive manufacturing’ OR ‘rapid prototyping’ OR stereolithography OR ‘patient specific model’ OR ‘printed model’ OR ‘navigation template’ OR ‘patient specific guide’) AND (spine OR spinal OR vertebra OR scoliosis OR kyphosis OR ‘pedicle screw’)

Screening method: All retrieved titles and abstracts were screened.

Scopus

Search strategy:

TITLE-ABS-KEY((“3D printing” OR “three-dimensional printing” OR “3D printed” OR “additive manufacturing” OR “rapid prototyping” OR stereolithography OR “patient-specific model*” OR “printed model*” OR “navigation template*” OR “patient-specific guide*”) AND (spine OR spinal OR vertebra* OR scoliosis OR kyphosis OR “pedicle screw”))

Screening method: All retrieved titles and abstracts were screened.

Web of Science

Search strategy:

TS=((“3D printing” OR “three-dimensional printing” OR “3D printed” OR “additive manufacturing” OR “rapid prototyping” OR stereolithography OR “patient-specific model*” OR “printed model*” OR “navigation template*” OR “patient-specific guide*”) AND (spine OR spinal OR vertebra* OR scoliosis OR kyphosis OR “pedicle screw”))

Screening method: All retrieved titles and abstracts were screened.

Google Scholar

Search strategy: "3D printing" spine OR "additive manufacturing" spine OR "rapid prototyping" spine OR stereolithography spine

Screening method: Results sorted by relevance were screened for each query.

Manual Reference Screening

Search strategy:

The reference lists of all included articles and relevant background reviews were manually screened to identify additional eligible studies.

Screening method: Potentially relevant titles identified from reference lists were assessed using the same inclusion and exclusion criteria as database-derived records.
